# Factors influencing adherence to antiretroviral therapy among HIV-infected adults in Cross River State, Nigeria: a cross-sectional study

**DOI:** 10.11604/pamj.2022.43.187.37172

**Published:** 2022-12-09

**Authors:** Anastasia Ikilishi Isika, Adamu Shehu, Tukur Dahiru, Izuchukwu Frank Obi, Afiong Oboko Oku, Muhammad Shakir Balogun, Aniekan Etokidem

**Affiliations:** 1 Department of Community Medicine, University of Calabar, Calabar, Cross River State, Nigeria,; 2 Department of Community Medicine, University of Calabar Teaching Hospital, Calabar, Cross River State, Nigeria,; 3 Nigeria Field Epidemiology and Laboratory Training Program, Abuja, Nigeria,; 4 Department of Community Medicine, College of Medicine, Ahmadu Bello University, Zaria, Nigeria,; 5 Department of Community Medicine, University of Nigeria Teaching Hospital, Enugu, Nigeria,; 6 Center for Translation and Implementation Research, College of Medicine, University of Nigeria, Nsukka, Nigeria

**Keywords:** Adherence, antiretroviral therapy, predictors of adherence, highly active antiretroviral therapy, Nigeria

## Abstract

**Introduction:**

improved access to antiretroviral therapy (ART) has significantly increased the survival and quality of life of HIV-infected persons. Strict adherence to antiretroviral therapy (ART) is crucial if viral suppression must remain optimal. We assessed predictors of adherence to ART among adult patients in Cross River State (CRS), Nigeria.

**Methods:**

a cross-sectional survey was conducted among 999 adult patients on ART in selected secondary and tertiary health facilities in CRS from January to June 2017. Respondents were recruited using multistage technique. Data were collected using a pre-tested interviewer-administered questionnaire. Adherence was defined as clients taking at least 95% of their pills in the last seven days. Multivariate analysis was performed to determine predictors of adherence at 5% level of significance.

**Results:**

majority (70.5%) of the respondents were females with a mean age of 43.7 ± 11.1 years. The self-reported adherence rate was 60.1%. The commonest reasons for non-adherence was client travelling out of home, being busy, forgetting and lack of food. The significant predictor identified in this study was being on first-line drugs (OR=3.677, 95% C.I=2.523-5.358), were 3 times more likely to have good adherence. Predictors of poor-adherence were alcohol intake (OR=0.382, 95% C.I=0.262-0.559), dosing medications (OR=0.502, 95% C.I=0.381-0.661), CD4 cell count ≥ 500 (OR=0.723, 95% C.I=0.543-0.964), poor attitude to HIV status and medication (OR=0.713, 95% C.I=0.512-0.994) and family support (OR=0.736, 95% C.I=0.544-0.995).

**Conclusion:**

adherence to ART among clients in this study was fair. Majority of the reasons for poor-adherence were client-related. There is need for targeted counselling to improve adherence.

## Introduction

With an estimated global prevalence of 0.8%, an estimated 38.4 million people living with HIV (PLHW) and 860,000 AIDS-related mortality globally in 2021, HIV continues to be a major public health problem [1,2]. Sub-Saharan Africa (SSA) accounts for over two-thirds of the global burden of HIV with Nigeria having one of the highest rates of new infection in SSA [3]. In 2020, 1.7 million people in Nigeria were living with HIV, with adult prevalence (15-45 years) of 1.3%, with estimated 86,000 new infections and 49,000 AIDS-related new deaths [4]. The south-south region of Nigeria, where the study site, Cross-River State is located, has the highest prevalence of HIV in Nigeria (5.5%) [5], while the prevalence in Cross River State is 1.7% [6].

Improved access to highly active antiretroviral therapy (HAART) has significantly improved quality of life, increased survival and reduced the risk of transmission of HIV to infants and sexual partners [7-9]. However, to obtain a successful treatment outcome, adherence to HAART needs to be greater than 95% otherwise; treatment failure that has been linked to increased mortality may result [10-13]. Several studies have measured adherence by expressing the number of doses taken as a percentage of the number of doses prescribed. Others have measured adherence through; patient self-report, pharmacy drug pickup and electronic methods (e.g. the Medication Event Monitoring System (MEMS) cap) [13-16]. Some factors that have been reported to be associated with good adherence in Nigeria include providing free medications, family and community support, availability of treatment-supporter, and family-based care if more than one family member is on HAART, amongst others [13,17]. Poor adherence to HAART has been linked to poor patient-caregiver relationship, high pill burden, forgetfulness, AIDS dementia complex, depression, and lack of patient education amongst others [13].

Some strategies that have been deployed overtime to improve adherence include treatment education for patients and partners, treatment-supporter involvement, peer health education, routine assessment and reinforcement of adherence during follow-up, directly observed therapy and addressing life-style factors [13,17]. A cross-sectional study conducted in a tertiary health facility in the same setting in 2013, reported a low adherence rate of 59.9% with participants citing being busy, forgetting, feeling depressed and traveling out as reasons for skipping their medications. Furthermore, predictors of good adherence were: perceived improved health status, reduced pill load and non-use of herbal remedies [16]. However, factors influencing adherence are known to be context-specific and change over time, hence the need to generate robust evidence on the factors influencing adherence to ART in our environment to guide interventions targeted at improving adherence. The study aimed to identify factors influencing adherence to ART among adult patients accessing care in secondary and tertiary healthcare facilities in urban and rural areas in Cross River State (CRS), Nigeria.

## Methods

**Study area and setting:** the study was conducted in Cross River State located in the southern part of Nigeria [18], with Calabar as its capital city [19]. The State shares boundaries with Akwa Ibom State to the South-West, Ebonyi and Abia to the West, Benue to the North, the Republic of Cameroon to the East and the Atlantic Ocean to the West. Cross River has 18 LGAs, with an estimated population of 3,674,951 (2015 estimate), projected from the 2006 census population at a growth rate of 2.9% [19]. The State is composed of several ethnic groups, which include the Efik, Ejagham, Yakurr, Bahumono, Bette, Yala, Igede, Ukelle, Utukwang and the Bekwarra [19]. The prevalence of HIV in the State dropped from 6.6% in 2014 to 1.7% in 2018 [20,21]. Cross River State, sandwiched between Benue and Akwa Ibom States, the two States with the highest prevalence of HIV in Nigeria, is a choice tourist destination with a large influx of visitors [6]. The four study facilities provide comprehensive HIV care (paediatric, adult and prevention of maternal to child transmission of HIV services) and run clinics from Monday through Friday. University of Calabar Teaching Hospital, the only tertiary healthcare facility in the State, has 5,800 patients accessing HIV care, of which more than 3,900 were on HAART; while General Hospital Calabar, had 11,057 patients with 3,294 on HAART.

**Study design:** a cross-sectional facility-based descriptive study.

**Study population:** we studied 999 adult (≥18 years) HIV-infected patients receiving treatment in three general hospitals (one from each senatorial district) and the only teaching hospital in the State.

**Eligibility criteria:** all the adult patients on HAART for at least three months prior to the study were eligible to participate. People living with HIV/AIDS (PLWHA) who were too sick, pregnant, had cognitive impairment or other disabilities like deafness, were excluded from the study.

**Sample size determination:** using the Leslie Kish formula for determination of minimum sample size based on the assumption of ART adherence rate of 59.9% among adult HIV-infected patients from a similar study [16], 5% precision, design effect of 2.4 and adjusting for a non-response rate of 10% non-response, the desired sample size was determined to be 985 rounded to 999 participants. This sample size was proportionately allocated to the study facilities.

**Sampling technique:** a multi-stage sampling involving three-stage was used to select participants for the study. The first stage involved selection of one local government area (LGA) each from the three senatorial districts in the State through simple random sampling (SRS). In the second stage (selection of study facilities), one general hospital was selected by SRS from each of the three selected LGA, while the only teaching hospital in the State was also included, giving a total of four selected facilities (General Hospital Calabar, General Hospital Ugep and General Hospital Obanliku representing the south, central and northern senatorial districts respectively, with University of Calabar Teaching Hospital included as the only teaching hospital in the State). Systematic random sampling was used in the third stage to recruit participants from the selected facilities based on proportionate allocation of sample size to each facility.

### Data collection

**Study instruments:** a pre-tested, semi-structured, interviewer-administered questionnaire adapted from a similar study [16] was used to collect information on participants´ socio-demographic characteristics, treatment history (when treatment commenced, type of HAART regimen), adherence profile and attitude to HAART.

### Data management and analysis

**Quantitative survey:** collected data uploaded to a secure server were exported in Microsoft Excel XLS format, cleaned in Excel spreadsheet and analysed with SPSS version 23. Frequencies, percentages, mean and standard deviations were used as appropriate in descriptive statistics. Adherence to HAART in the seven days preceding the interview was measured by self-report. The questions were adapted from brief medication questionnaire self-report tool for screening adherence and barriers to adherence [16]. The degree of adherence from patient self-report was estimated using the following formula [22].


$$\%\text{ adherence over last 7 days=}\frac{\#\text{ Doses should have taken - \# Missed doses}}{\#\text{ Doses should have taken}}\times 100\%$$

For the purpose of the study, adherence score of 95% and above represented good adherence while scores of less than 95% were rated as poor adherence. At bivariate analysis, association between HAART adherence and independent variables such as socio-demographic and treatment characteristics were determined using Chi-square tests with corresponding p-values. Variables that had a p-value of ≤ 0.2 at bivariate analysis were entered into the logistic regression model to determine the predictors of HAART adherence. The results of the logistic regression were reported using odds ratios and 95% confidence intervals. All statistical analyses were performed at 5% level of significance.

**Ethical considerations:** ethical approval for this study was obtained from the Health Research Ethics Committees of University of Calabar Teaching Hospital (reference: UCTH/HREC/33/519) and the State Ministry of Health (reference: RP/REC/2016/422). Written informed consents were obtained from the participants after the details of the study and its voluntariness was explained to them. Safety and confidentiality of collected data was ensured throughout the study.

## Results

The mean age of the 999 study participants was 43.7 ± 11.1 years. Majority 704 (74.5%) were females, married 466 (46.6%), attended secondary education 436 (43.6%), were self-employed 482 (48.2%), and earned less than ₦ 20,000 (USD 50) monthly 565 (56.5%) (Table 1). In addition, majority 842 (84.2%) of respondents were on first line drugs while 515 (51.6%) were on once daily regimen. Adherence pattern revealed that a little over half, 541 (54.1%) of the respondents did not miss any tablet in the last week, while 439 (43.9%) missed 1- 2 tablets. Majority of the respondents 743 (74.4%) refill their drugs every 2 months, while 160 (16.0%) refills monthly. Additionally, 591 (59.2%) of the respondents spent ≤ ₦ 500 (USD 1.1) on transportation to the health facility (Table 2).

**Table 1:** socio-demographic characteristics of respondents on HAART, Cross River State, 2017

Variables	Frequency (N =999)	Percentage (100%)
**Age distribution (years)**		
≤ 29	98	9.9
30 - 39	268	26.8
40 - 49	307	30.7
≥ 50	326	32.6
Median age (years) ± SD	43.7 ± 11.1 years	
**Sex**		
Male	295	29.5
Female	704	70.5
**Religion**		
Christianity	990	99.1
Islam	8	0.8
Traditional	1	0.1
**Marital status**		
Single	224	22.4
Married / cohabiting	466	46.6
Divorced / Separated	70	7.1
Widowed	239	23.9
**Highest level of education**		
None	93	9.3
Primary	236	23.6
Secondary	436	43.6
Tertiary	234	23.5
**Employment status**		
Unemployed	227	22.7
Paid employment	210	21.0
Self-employed	482	48.2
Others (retired, student)	80	8.1
**Average monthly income**		
≤ N 9000	353	35.3
N 10,000 - 19,000	212	21.2
N 20,000 - 29,000	131	13.1
≥ N 30,000	303	30.4
**Place of residence**		
Urban	583	58.4
Rural	416	41.6
**Alcohol**		
Consumed	188	18.9
Never consumed	811	81.1
**Smoke cigarette**		
Yes	25	2.5
No	974	97.5
**Disclosure**		
Yes	913	91.4
No	86	8.6
**Support from family**		
Yes	691	69.2
No	308	30.8

**Table 2:** clinical characteristics of respondents on HAART, Cross River State, 2017

Variables	Frequency (N=999)	Percentage (100%)
**Duration of treatment (months)**		
<12 months	82	8.2
12-24 months	144	14.4
>24 months	773	77.4
**HAART regimen**		
First line	842	84.3
Second line	157	15.7
**HAART dosing**		
Once	515	51.6
Twice	484	48.4
**Number of pills per day**		
1	497	49.8
2	495	49.5
≥3	7	0.7
**Number of tablets missed/week**		
0	541	54.2
1-2	439	43.9
≥3	19	1.9
**Fluid and food restrictions**		
No	995	99.6
Yes	4	0.4
**Encountered side effects**		
No	956	95.7
Yes	43	4.3
**Drug refill**		
≤1 monthly	160	16.0
2 monthly	743	74.4
3 monthly	96	9.6
**Transport cost to HF**		
≤ ₦ 500 (USD 1.1)	591	59.2
> ₦ 500 (USD 1.1)	408	40.8
**CD4 count**		
< 500	592	59.3
≥ 500	407	40.7

HAART: highly active antiretroviral therapy; HF: health facility

**Figure 1:**
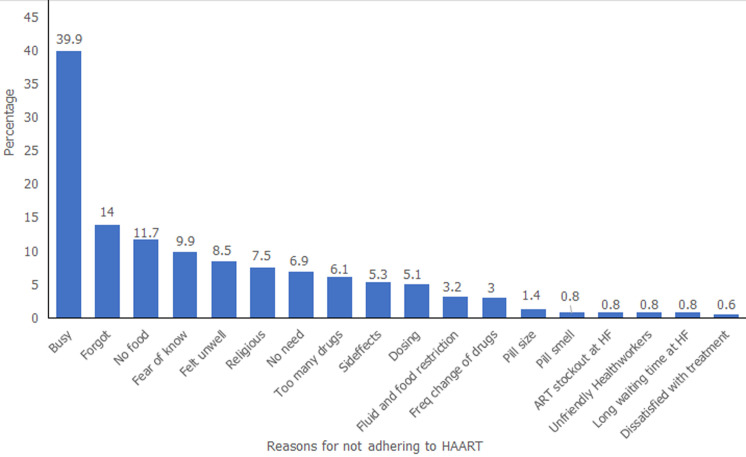
respondents’ reasons for missing HAART, Cross River State Nigeria, 2017

Out of 999 participants, 60.1% had good adherence, defined as taking at least 95% of their pills during the previous 7 days prior to the study. The most common reasons given by respondents for not adhering to HAART were mostly client related. They include being busy (39.9%), forgot (14.0%), no food (11.7%), fear of known (9.9%), felt unwell (8.5%). The least common reasons for not adhering to HAART were mostly provider related. These include dissatisfaction with treatment (0.6%), long clinic waiting time (0.8%), unfriendly health workers (0.8%), and pill having smell (0.8%) (Figure 1).

Bivariate analysis revealed that respondents who resided in urban settlements (χ^2^= 4.314, p=0.038), had formal education (χ^2^= 5.825, p=0.016), did not take any form of alcohol (χ^2^= 21.282, p=<0.001), did not receive any form of family support (χ^2^= 5.019, p=0.025), those on first line ART (χ^2^= 46.199, p=<0.001), once daily regimen (χ^2^= 27.148, p=<0.001), regimen had no food restrictions (Fisher’s Exact Test = 0.000, p=0.025), did not experience side-effects (χ^2^= 4.535, p=0.033), and whose CD4 cell count were ≥ 500 (χ^2^= 9.591, p=0.002) were more likely to have good adherence to HAART compared to their counterparts (Table 3).

**Table 3:** association between respondents’ socio-demographics and adherence to HAART

Variables	Adherence			
	Good adherence	Poor adherence	χ2	P-value
**Age**				
<43	319(61.9)	196(38.1)	1.569	0.210
≥43	281(58.1)	41(41.9)		
**Sex**				
Male	182(61.7)	113(38.3)	0.466	0.495
Female	418(59.4)	286(40.6)		
**Marital status**				
Married / cohabiting	286(61.4)	180(38.6)	1.306	0.728
Separated / divorced	39(55.7)	31(44.3)		
Widowed	145(60.7)	94(39.3)		
Single	130(58.0)	94(42.0)		
**Employment status**				
Employed	475(61.5)	297(38.5)	3.054	0.081
Not employed	125(55.1)	102(44.9)		
**Type of settlement /resident**				
Urban	366(61.0)	217(54.4)	4.314	0.038
Rural	234(39.0)	182(45.6)		
**Level of education**				
No formal education	45(48.4)	48(51.6)	5.825	0.016
Formal education	555(61.3)	351(38.7)		
**Comorbidities**				
No	404(59.0)	281(41.0)	1.064	0.302
Yes	196(62.4)	118(37.6)		
**Alcohol**				
No	515(63.5)	296(35.9)	21.282	<0.001
Yes	85(45.2)	103(54.8)		
**Disclosure**				
No	54(62.8)	32(37.2)	0.293	0.589
Yes	546(59.8)	367(40.2)		
**Family support**				
No	201(65.3)	107(34.7)	5.019	0.025
Yes	399(57.7)	292(42.3)		
**HAART combination**				
First line	544(64.6)	298(35.4)	46.199	<0.001
Second line	56(35.7)	101(64.3)		
**Number of pills per day**				
≤2	596(60.1)	396(39.9)	FET 0.000	1.000
>2	4(57.1)	3(42.9)		
**HAART dosing**				
Once daily	269(52.2)	246(47.8)	27.148	<0.001
Twice daily	331(68.4)	153(31.6)		
**Food and water restriction**				
No	600(60.3)	395(39.7)	FET 0.000	0.025
Yes	0(0.0)	4(100)		
**Encountered side effects**				
No	512(61.5)	320(38.5)	4.535	0.033
Yes	88(52.7)	79(47.3)		
**CD4 count**				
<500	332(56.1)	260(43.9)	9.591	0.002
≥500	268(65.8)	139(34.2)		

HAART: highly active antiretroviral therapy; FET: Fisher’s exact test

On multivariate analysis, respondents who were on first line drugs (OR= 3.68, 95% C.I = 2.52 -5.36), were 3 times more likely to have good adherence. However, respondents who drank any form of alcohol (OR=0.38, 95% C.I=0.26-0.56), were on twice-daily medication (OR=0.50, 95% C.I=0.38-0.66), those who had CD4 cell count ≥ 500 (OR=0.72, 95% C.I=0.54-0.96), had poor attitude towards their HIV-status and medication (OR=0.71, 95% C.I=0.51-0.99) as well as those who had any family support (OR=0.736, 95% C.I=0.54-0.99) were less likely to have good adherence (Table 4).

**Table 4:** association between treatment profile and adherence to HAART among respondents

Characteristic	Odds ratio	95% confidence interval		p-value
		Lower	Upper	
**Median age**				
< 43	1	0.779	1.443	0.724
≥ 43	1.056			
**Type of residence**				
Rural	1	0.674	1.306	0.714
Urban	0.938			
**Educational status**				
Formal education	1	0.801	2.150	0.280
No formal education	1.313			
**Drink alcohol**				
No	1	0.262	0.559	<0.001
Yes	0.382			
**HAART combination**				
Second line	1	2.523	5.358	<0.001
First line	3.677			
**HAART dosing**				
Once daily	1	0.381	0.661	<0.001
Twice daily	0.502			
**Side effects**				
No	1	0.547	1.129	0.192
Yes	0.786			
**Food and fluid restriction**				
No	1	0.000		0.999
Yes	0.000			
**CD4 count**				
< 500	1	0.543	0.964	0.027
≥ 500	0.723			
**Family support**				
No	1	0.544	0.995	0.046
Yes	0.736			
**Co-morbidity**				
No	1	0.659	1.330	0.714
Yes	0.936			

HAART: highly active antiretroviral therapy

## Discussion

This study assessed factors influencing adherence to HAART among adults infected with HIV in Cross River State, Nigeria. Only three-fifth of the respondents had good adherence. This adherence level is suboptimal considering the fact that the respondents were patients who came for refill. This suggests that adherence could even be worse among those who could not keep clinic appointment. This reported low adherence level is just a slight improvement over 59.9% adherence level reported by a previous study in the State [16]. Furthermore, other studies conducted in public and private facilities in Nigeria have reported adherence rates ranging from of 58% - 95.3% [14,17,20,21,23,24] in public facilities. The disparity of adherence rates across studies may be due to different geographical locations and may be due to variations in how adherence was measured. Our study-assessed adherence using self-report and timing while some of the other studies used pill count. Sub-optimal adherence to HAART as found in our study calls for urgent interventions targeted at improving adherence.

The most common reasons given for poor-adherence were client related. About two-fifth of the respondents cited being busy as one of the major reasons for not adhering to medications. This finding is consistent with the findings from other studies. Studies conducted in Ethiopia revealed that the reasons given for missing drugs were being away from home and being busy with other things and that those whose medication-taking time interfered with their daily routines had more than fifteen times higher odds of non-adherence compared to those whose medication schedules did not interfere with their daily activities [25-27]. Therefore, reducing the pill burden and frequency of taking the drugs could improve adherence. The implication of this is that drug manufacturers should formulate drugs where a single tablet should contain all the major ingredients and the dosage frequency reduced to once daily medication in order to optimize adherence. Other reasons for poor-adherence include forgot, not having food, fear of being known, felt unwell, no need, too many drugs, fear of side effects, dosing, amongst others. These reasons have been reported by other studies as contributing to non-adherence [15,16,27-29]. Only few respondents mentioned provider related factors like long waiting time, dissatisfaction with treatment, unfriendly health worker attitude and unavailable drugs as reasons for non-adherence. Health system and provider-related factors not being cited much as reasons for non-adherence to HAART in this study suggests high quality service delivery at the study facilities.

This study also revealed that respondents who took alcohol in any form were less likely to have good adherence to their medication. This is in keeping with a study in Enugu, Nigeria which revealed that respondents who did not take alcohol had approximately four times the odds of being adherent to their medications when compared to those who took alcohol [15]. Other studies have reported similar findings in other parts of Nigeria [15,29] and Ethiopia [30]. The use of alcohol and other addictive substances have been linked with poor adherence to HAART. This could be due to the tendency of these substances to cause forgetfulness, poor organization and diversion of monetary resources [17,24,30-32]. It is therefore important that patients on HAART be counselled to avoid alcohol intake and use of other addictive substances that may cause memory impairment.

Furthermore, respondents who were on twice-daily HAART regimen were less likely to have good adherence. Similar findings have been reported by studies in other parts of Nigeria [17], Peru [33] and the United States of America [31]. This is understandable because people tend to be busy and may find it difficult to incorporate their drug schedule into their daily activities. Therefore, reducing HAART dosing frequency and pill burden should always be the target. Closely linked to pill-burden is the finding that respondents on first-line drugs were three times more likely to have good adherence compared to those on second-line drugs. This has also been reported by studies from other parts of Nigeria [24]. The study also found that respondents whose CD4 cell counts were above 500 cells/mm^3^ were less likely to have good adherence. This may be attributed to the reduced likelihood of illness in such patients; hence, the feeling of being healthy may make them not see the need for the drugs. This might lead to relapse and drug failure. It is therefore important to always educate the patients on the need to continue adhering to their medications in order to sustain the gains achieved. Surprisingly, in contrast with reports from other studies [34], this study found that respondents who had no family support had better adherence compared to those with family support. The reason behind this finding is unclear and calls for further investigation.

This study has provided insight on some important context-specific factors influencing adherence to HAART in a resource-limited setting. However, the study has a few limitations. This study being a cross-sectional study which relied on respondents´ ability to accurately recall how they took their HAART, may have been prone to recall bias. To make it easier for clients to accurately remember their experience, we limited recall to the past seven days. Furthermore, clients´ self-report of adherence may have been prone to social desirability bias whereby respondents report good adherence in order to please the investigators. We however reassured participants that their response would in no way affect their treatment in the facility. We also ensured they had privacy during questionnaire administration with no health facility worker around. The findings from this study are generalizable to similar populations especially in low resource settings.

## Conclusion

This study showed a suboptimal adherence level among clients accessing care in facilities in Cross River State, Nigeria. The main predictors of good adherence include not drinking any form of alcohol, being on once-daily medication, being on first-line drugs, having no family support, as well as having a CD4 count above 500 cell/mm^3^. The authors therefore recommend tailored adherence enhancing strategies targeted at identified barriers of good adherence.

### What is known about this topic

- Adherence to ART among HIV-infected patients is essential for optimal clinical outcomes;
- Adherence to ART varies in different settings and factors influencing adherence also vary in different settings.

### What this study adds

- Adherence to ART among adults infected with HIV is low in Cross River State, Nigeria;
- The major reasons for non-adherence were client related and include feeling depressed, travelling out of home, being busy and forgetting;
- Predictors of good adherence to ART include not drinking any form of alcohol, being on once-daily medication, being on first-line drugs, having no family support, as well as having a CD4 count ≥500.
